# Targeting the gp130/STAT3 Axis Attenuates Tumor Microenvironment Mediated Chemoresistance in Group 3 Medulloblastoma Cells

**DOI:** 10.3390/cells11030381

**Published:** 2022-01-23

**Authors:** Lakshana Sreenivasan, Ling Vicky Li, Pascal Leclair, Chinten James Lim

**Affiliations:** 1 Michael Cuccione Childhood Cancer Research Program, BC Children’s Hospital Research Institute, Vancouver, BC V5Z 4H4, Canada; lsreenivasan@bcchr.ca (L.S.); lli2@bcchr.ca (L.V.L.); pleclair@bcchr.ca (P.L.); 2 Department of Medicine, University of British Columbia, Vancouver, BC V5Z 4H4, Canada; 3 Department of Pathology and Laboratory Medicine, University of British Columbia, Vancouver, BC V5Z 4H4, Canada; 4 Department of Pediatrics, University of British Columbia, Vancouver, BC V5Z 4H4, Canada

**Keywords:** interleukin-6, JAK1, STAT3, medulloblastoma, drug resistance, combination therapy

## Abstract

Medulloblastoma (MB) is the most common malignant pediatric brain tumor. Of the four molecular subgroups, Group 3 MB is the most aggressive and has the worst prognosis. To understand the origins of chemoresistance involving IL-6/STAT3 signaling, we used in vitro co-culture systems to investigate the contribution of microglia as a brain tumor microenvironment cellular source of paracrine cytokines that promotes acquired drug resistance in Group 3 MB. MB cells subjected to co-culture with microglia exhibited increased expression of phosphorylated JAK1 and STAT3, which was correlated with enhanced resistance to vincristine. We found that both microglia and MB cells co-cultured with microglia secreted significant quantities of IL-6, indicating that IL-6 is a paracrine and autocrine cytokine able to initiate and sustain STAT3 activity in MB cells. Surprisingly, IL-6R^−/−^ MB cells, which cannot respond to exogenous IL-6 stimuli, were responsive to microglia co-culture induced activation of STAT3 and chemoresistance. Subsequently, we found that MB cells conditioned in vitro with the IL-6 family cytokines, IL-6, OSM, LIF, or IL-11, exhibited enhanced JAK1/STAT3 activity and chemoresistance. Intriguingly, MB cells conditioned with any one of the IL-6 family cytokine secreted multiple IL-6 family cytokines, implicating a feedback network involving multiple cytokines. The IL-6 family cytokine receptors share a common signal transducing β-subunit, gp130, which may be targeted to mitigate tumor chemoresistance. We showed that microglia co-culture failed to induce chemoresistance of gp130^−/−^ MB cells, and that combination treatment using gp130 inhibitors, or with the JAK inhibitor ruxolitinib, effectively overcame the observed resistance to vincristine in gp130 expressing MB cells. Our in vitro studies highlight the gp130/JAK/STAT pathway as a therapeutic target in combating acquired treatment resistance in Group 3 MB.

## 1. Introduction

Medulloblastoma (MB) is a high-grade pediatric brain malignancy comprising 10% of all childhood brain tumors [1,2]. In the last decade, high-throughput transcriptomics, genome-wide methylation arrays and advanced molecular tools have been utilized to classify MB into four distinct subgroups: WNT, SHH, Group 3, and Group 4 [3,4,5,6,7]. The molecular classification has facilitated stratification of MB tumors into distinct risk groups and calls for a specific treatment paradigm for each molecular subgroup [8]. Of the four subgroups, Group 3 MB, which had the lowest 5-year survival rate at about 50%, was also the most aggressive, with nearly half of patients presenting with metastatic disease at initial diagnosis [8,9]. Combined with a lack of defined molecular pathways for targeting, refractory and relapsed Group 3 MB remain the most challenging to manage clinically, necessitating pre-clinical studies to understand the mechanisms of aggressive tumor behavior.

Cytotoxic chemotherapy remains one of the principal modes for cancer treatment. Vincristine, cisplatin, lomustine, and cyclophosphamide are some of the agents approved for the clinical treatment of MB patients [9,10]. However, the effectiveness of cytotoxic chemotherapy is often limited by acquired drug resistance or intolerable toxicity effects [11,12]. In addition, aggressive therapy also leads to severe long-term complications and the development of secondary tumors in individuals with MB [9]. Hence, a more effective treatment approach is required to alleviate the therapeutic burden and to overcome the development of drug resistance in MB.

The interleukin 6 (IL-6) family cytokines initiate classic signaling by binding to either a glycoprotein 130 (gp130) homodimer receptor complex (e.g., IL-6R, IL-11R) or to a gp130 heterodimer receptor complex (e.g., LIFR, OSMR) [13]. This cytokine–receptor complex triggers phosphorylation and activation of the gp130 associated Janus kinase (JAK), in turn leading to recruitment, phosphorylation, and activation of cytoplasmic signal transducer and activator of transcription (STAT), which then translocates into the nucleus and mediates transcription [14].

The IL-6 family cytokines play crucial roles in early development, maintaining normal physiological homeostasis and inflammatory responses [15,16]. A feature of IL-6 family cytokine receptors is their shared common signal transducing receptor β-subunit, gp130 (also known as IL-6ST or CD130), which is ubiquitously expressed in all human cells [13,17,18,19]. Dysregulation of gp130-mediated signaling has been implicated in numerous diseases including autoimmune diseases, chronic inflammatory diseases, and cancer [13,20]. It is also known that downstream JAK-mediated STAT3 signaling in both tumor cells and immune cells contributes to tumorigenesis, whose activity has also been associated with drug resistant tumors [13,21,22]. Importantly, the IL-6/JAK/STAT3 pathway is amenable for therapeutic targeting. Most prominently used is ruxolitinib, a potent but selective JAK1/2 inhibitor that has been used to treat myeloproliferative disorders and acute leukemias by inhibiting cell growth [23,24,25]. Recent studies have also demonstrated the efficacy of inhibitors that target gp130 signaling in multiple malignancies. This includes bazedoxifene, an FDA-approved selective estrogen receptor modulator (SERM) repurposed as a gp130 inhibitor [26,27] as well as SC144, another gp130 inhibitor used in preclinical studies [28]. Ultimately, these agents prevent the phosphorylation and activation of STAT3 as a key oncogenic pathway.

The tumor microenvironment (TME) is a fundamental regulator of cancer progression that also governs therapeutic efficacy in primary and metastatic brain malignancies [29]. The presence of IL-6 family cytokines in the brain TME has gained interest for its newly recognized role in central nervous system (CNS) homeostasis and pathogenesis of CNS diseases [30,31,32]. In previous work, we uncovered an autocrine feedback loop involving sustained IL-6/STAT3 signaling driving rapid conversion of chemosensitive Group 3 MB cells into chemoresistant variants [33]. Importantly, chemosensitive MB secreted little IL-6, raising the question of the cellular source for the initiating cytokine stimuli. We postulated that certain immune cells within the brain TME could initiate paracrine cytokine signaling. This led to our focus on the role of microglia, tissue-resident macrophages of the CNS known to secrete soluble factors including IL-6, which can facilitate tumor growth and survival in a bidirectional crosstalk manner [29,34]. Here, we investigated the role of microglia as a source of cytokines that can promote STAT3 activity and chemoresistance to vincristine in Group 3 MB via paracrine signaling. In addition, we also evaluated the potential of targeting the IL-6/STAT3 signaling axis using inhibitors against gp130 and JAKs as an improved therapeutic strategy to circumvent drug resistance in Group 3 MB. An improved understanding of the tumor-promoting role of microglia and cytokines present in the brain TME will aid in identifying the key survival pathways and development of potential therapeutics to combat drug resistance and disease progression of Group 3 MB.

## 2. Materials and Methods

### 2.1. Cells and Tissue Culture

MED-MEB-8A [35] (herein known as Med8A-S cells) was maintained in DMEM (Sigma, Oakville, ON, Canada) with 10% fetal bovine serum (FBS, Invitrogen, Burlington, ON, Canada). D283 Med (ATCC HTB185™) and D341 Med (ATCC HTB187™) were maintained in 10% FBS EMEM (Sigma, Oakville, ON, Canada) and 20% FBS EMEM, respectively. Human microglia cell line, HMC3 (ATCC CRL-3304™) was maintained in 10% FBS EMEM (Sigma, Oakville, ON, Canada). All media were also supplemented with 1% penicillin–streptomycin and non-essential amino acids (Invitrogen).

### 2.2. Co-Culture System and Cytokine Conditioning

To condition cells with cytokines (denoted as IL-6+, LIF+, OSM+ and IL-11+), each cell line was cultured for four weeks in media with 2 ng/mL recombinant human IL-6, LIF, OSM, and IL-11 (Genscript, Piscataway, NJ, USA), respectively. Following conditioning, cells were cultured for two weeks without cytokines prior to use in experiments.

MB cells were also conditioned to microglia using a no-contact co-culture system. Target MB cells were plated at a density of 10^5^ cells/well in a 6-well plate, while HMC3 microglia were plated at a density of 10^4^ cells within a Transwell insert (Greiner ThinCert, ThermoFisher, Burlington, ON, Canada). The co-culture was initiated by placing the Transwell insert onto the target cells in fresh media with no added cytokines. The insert was removed after three days, and the conditioned MB cells rinsed and replenished with fresh media for another three days (wean-off stage). Cells were either harvested for drug treatment or protein analysis, and in other experiments, the cell free media supernatant was harvested for the analysis of secreted cytokines.

### 2.3. Drug Treatment and Cell Viability Assays

Cells were seeded at a density of 10^5^ cells/well in 96-well plates for 16 h to facilitate adhesion, before the addition of vincristine (Sigma, Oakville, ON, Canada) at various concentrations. After 48 h, the fluorometric reagent Cell Titer Blue (Promega, Madison, WI, USA) was added and fluorescence (560_Ex_/590_Em_) measured on a spectrophotometer (PE Enspire, Waltham, MA, USA) after 4 h. In some experiments involving microglia co-culture conditioning, MB cells were treated with ruxolitinib, bazedoxifene, or SC144 (Selleckchem, Houston, TX, USA) during the wean-off three day period, prior to replating cells in 96-well plates for combination treatment of the same agent with vincristine. All assays were conducted as three replicates per treatment condition.

### 2.4. Western Blots

Cells were lysed in PN buffer (PN is 10 mM PIPES, 150 mM sucrose, 50 mM NaCl, 50 mM NaF, 40 mM Na_4_P_2_O_7._10H_2_O, 1 mM Na_3_VO_4_, 1% Triton X-100, and complete protease inhibitors (Sigma, Oakville, ON, Canada)). Total protein (30 µg) was separated by SDS-PAGE, transferred to nitrocellulose, and blots blocked in 5% bovine serum albumin (BSA, ThermoFisher) in TBS-T (TBS-T is 50 mM TrisHCl pH 8, 150 mM NaCl, 0.1% Tween 20) for 1 h at 22 °C, then incubated overnight at 4 °C with primary antibodies diluted in blocking buffer. Blots were then incubated with fluorophore-conjugated goat anti-mouse or -rabbit antibodies (Dylight 680 or 800, ThermoFisher) in 2% non-fat milk in TBS-T, and imaged on the Licor Odyssey (Lincoln, NE, USA).

The following primary antibodies were used: pY705-STAT3, STAT3, pY1034/1035-JAK1, JAK1, pY1008-JAK2, JAK2, pY1054/1055TYK2, TYK2 (Cell Signaling Technologies, Danvers, MA, USA); and GAPDH (Biolegend, San Diego, CA, USA). In some experiments, cells were treated with IL-6, LIF, OSM, or IL-11 (Genscript) at the indicated concentrations and time prior to the preparation of cell lysates.

### 2.5. Plasmids and CRISPR

Guide RNA (gRNA) mediated CRISPR-Cas9 gene editing was used to generate null cell lines. Derivation of the Med8A-based IL-6R^−/−^ cell line was as described previously [33]. To target exon 2 of *gp130*, the 5′ GGTGAACTTCTAGATCCATG 3′ guide sequence was cloned into pX458 (Addgene #48138, Watertown, MA, USA). Med8A-S cells were transfected with this plasmid using Lipofectamine 2000 (ThermoFisher), and clonally flow-sorted the next day into 96-well plates (FacsAria, BD Biosciences, Mississauga, ON, Canada). Screening for gp130 null clones was done by flow cytometry. To identify indel mutations within the targeted genomic loci, we sequenced a genomic PCR amplicon using the following primers: 5′ GTTGACGTTGCAGACTTGG 3′ (Fwd) and 3′ CCTTCCACCATCCCACTCAC 5′ (Rev). CLC Main Workbench (Qiagen, Valencia, CA, USA) was used to perform sequence alignments of the CRISPR mutant and parental strain.

### 2.6. Flow Cytometry

To evaluate cell surface gp130, IL-6R, OSMR, LIFR, and IL11R expression, cells disaggregated with trypsin-EDTA were washed with phosphate buffered saline and probed with anti-human IL-6Rα antibody (R&D Systems, Toronto, ON, Canada), OSMRβ (ThermoFisher), IL-11Rα (ThermoFisher), LIFRα (Bioss, Woburn, MA, USA), or gp130 (Biolegend) followed by Dylight 488-conjugated secondary antibody (ThermoFisher). Flow cytometry was performed on the Accuri C6 (BD Biosciences) and post-acquisition analysis using FlowJo (BD Biosciences). Fluorescence activated cell sorting of CRISPR generated cells was conducted in the BCCHRI Flow Core Facility (FacsAria, BD Biosciences).

### 2.7. Secreted Cytokine Assays

Secreted IL-6, IL-11, LIF, and OSM in the media supernatant of cultured cells was quantified using a LEGEND MAX Human IL-6 ELISA Kit (Biolegend #430507), Human LIF ELISA Kit (Raybiotech #ELH-LIF-1, Peachtree Corners, GA, USA), Human OSM ELISA Kit (Raybiotech #ELH-OSM-1), and Human IL-11 ELISA Kit (Raybiotech #ELH-IL11-1), as per the manufacturer’s instructions. Absorbance at 450 nm was measured on a microplate reader (PE Enspire).

### 2.8. Statistical Data Analysis

All data are representative of at least three independent experiments. Graphs were plotted and statistical significance calculated using Prism (GraphPad, San Diego, CA, USA), with *** *p*  <  0.001, ** *p*  <  0.01, * *p*  <  0.05, ns—not significant. The statistical tests used is as indicated in the figure captions.

## 3. Results

### 3.1. MB Cells Co-Cultured with Microglia Exhibit Increased STAT3 Activity and Chemoresistance

**Figure 1:**
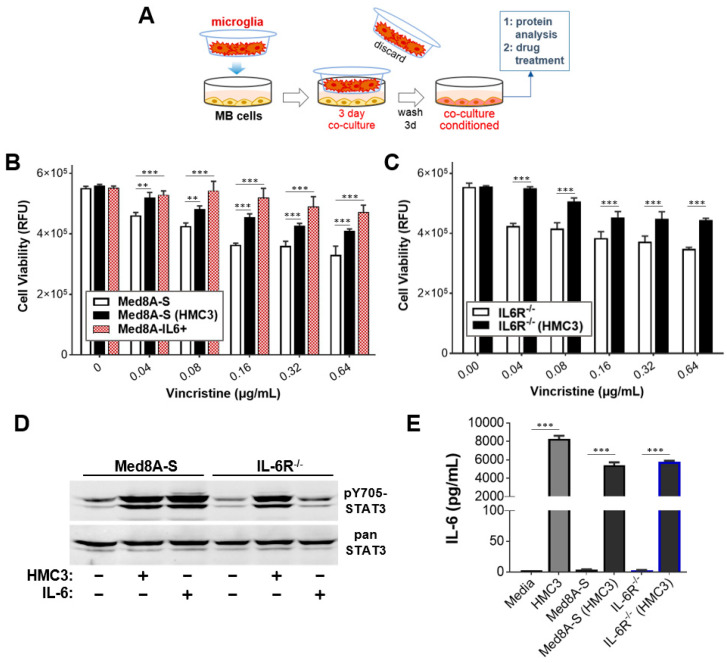
Exposure to microglia render Med8A cells chemoresistant concurrent with enhanced STAT3 activity. (**A**) Schematic of the co-culture system used to study the effect of microglia on Group 3 MB cell lines. MB cells (e.g., Med8A-S) are plated in the bottom well, while microglia (HMC3) cells are plated within the Transwell insert. The 1 µm pores of the insert enable media exchange between the two cell populations without direct contact. The insert is discarded after three days, the MB cells were washed, and cultured a further three days in fresh media. Following this conditioning, cells were harvested for drug treatment assays and/or protein analysis, while the cell-free conditioned media were analyzed for secreted cytokines. (**B**,**C**) Cell viability assay to assess the sensitivity of Med8A-S and IL-6R^−/−^ cells to vincristine with or without co-culture with HMC3 cells. In (**B**), Med8A-IL6+ cells refer to Med8A-S cells conditioned with IL-6 for four weeks (as detailed in Methods). As plotted is the mean  ±  SD of an experiment performed in triplicate, representative of at least three independent experiments. *** *p* <  0.001, ** <  0.01, two-way ANOVA with Bonferroni’s multiple comparison test. (**D**) Western blot analysis of lysates of Med8A-S or IL-6R^−/−^ cells for pY705-STAT3 and STAT3 expression, with or without HMC3 co-culture or stimulation with IL-6. (**E**) Analyses for secreted IL-6 in culture supernatant of the indicated cells, without or with HMC3 co-culture. Plotted is the mean  ±  SD of the triplicate assays; *** *p* <  0.001, two-way ANOVA with Tukey’s multiple comparison test.

Microglia found in the brain TME is a potential source of paracrine cytokines that can promote acquired chemoresistance in Group 3 MB cells [29]. To assess this, we used a no-contact co-culture system whereby Group 3 MB cells are continuously exposed to factors secreted by the human microglia cell line, HMC3, contained within a Transwell insert (Figure 1A). Med8A-S cells that had been co-cultured with HMC3, denoted as Med8A-S(HMC3) cells, demonstrated significant resistance to vincristine treatment when compared to non-co-cultured Med8A-S cells (Figure 1B). However, Med8A-S cells that had been conditioned with exogenous IL-6, denoted as Med8A-IL6+ cells, appeared to be more chemoresistant when compared to Med8A-S(HMC3) cells (Figure 1B). This difference is likely attributable to the duration of conditioning, where HMC3 co-culture was for three days while IL-6 conditioning was for four weeks. In our previous study, we showed that IL-6R^−/−^ cells failed to respond to IL-6 conditioning and remained susceptible to vincristine treatment [33]. Interestingly, IL-6R^−/−^ cells co-cultured with HMC3 were found to exhibit significant chemoresistance to vincristine (Figure 1C), suggesting that soluble factors in addition to IL-6 released by microglia may promote chemoresistance in both Med8A-S and IL-6R^−/−^ cells.

We evaluated whether the observed chemoresistance of MB cells upon co-culture with microglia can be correlated with changes in STAT3 activity. Both Med8A-S and IL-6R^−/−^ cells that were co-cultured with HMC3 exhibited elevated levels of pY705-STAT3 expression (Figure 1D). In contrast, bolus IL-6 treatment resulted in increased pY705-STAT3 expression in Med8A-S cells, but not in IL-6R^−/−^ cells lacking the IL-6 receptor. To assess whether secreted IL-6 contributes to sustained signaling, we measured IL-6 in the tissue culture supernatant of HMC3 and Med8A cells, either alone or when co-cultured together. As shown in Figure 1E, HMC3 cells secreted high levels of IL-6, which is likely to act in paracrine fashion in a co-culture to activate IL-6/STAT3 signaling in Med8A-S cells. Indeed, Med8A-S cells that had been co-cultured with HMC3 also secreted IL-6. We also found that IL-6R^−/−^ cells co-cultured with HMC3 also secreted significant amounts of IL-6 despite lacking IL-6R expression (Figure 1E), strongly suggesting that IL-6 secretion by MB cells could be triggered by cytokine–receptor pairs in addition to the IL-6/IL-6R combination. These results demonstrate that HMC3 microglia is a source of stimulatory cytokines including IL-6, which acts in paracrine fashion to promote STAT3 signaling in Med8A MB cells, in turn, promoting further secretion of IL-6 and possibly other cytokines that can act in an autocrine manner and enable tumor cells to gain chemoresistance.

### 3.2. JAK Inhibitor Ruxolitinib Diminishes Microglia Co-Culture Mediated Chemoresistance of MB Cells

**Figure 2:**
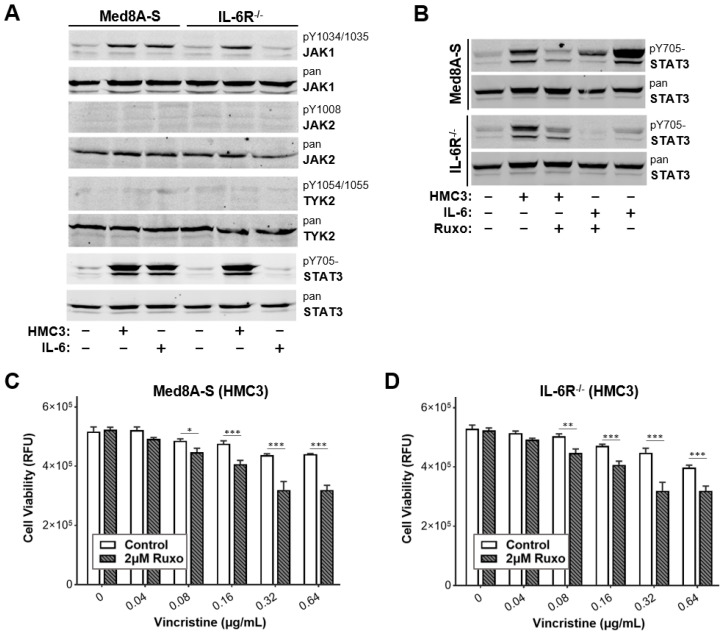
JAK inhibitor suppresses STAT3 activity and overcomes microglia-induced chemoresistance of MB cells. (**A**) Med8A-S or IL-6R^−/−^ cells were untreated, co-culture treated with HMC3, or treated with bolus 5 ng/mL IL-6, as indicated. As shown are cell lysate immunoblots for pJAK1, JAK1, pJAK2, JAK2, pTYK2, TYK2, pY705-STAT3, and STAT3 expression. (**B**) In addition to HMC3 co-culture or bolus IL-6 stimulation, some cells were also treated with 2 µM ruxolitinib for 20 min prior to cell lysate preparation for immunoblot analyses of pY705-STAT3 and STAT3 expression. (**C**,**D**) Cell viability assay to assess the sensitivity to vincristine of Med8A-S or IL-6R^−/−^ cells that had been co-cultured with HMC3, with or without co-treatment with 2 µM ruxolitinib. Plotted is the mean  ±  SD of triplicate assays, representative of three independent experiments. *** *p*  <  0.001, ** < 0.01, * < 0.05, two-way ANOVA with Bonferroni’s multiple comparison test.

Janus kinases (JAK1, JAK2 and TYK2) are crucial signal transducers that link cytokine-bound receptors to the phosphorylation of STAT proteins [14]. An initial western blot analysis of Med8A-S cells revealed that bolus IL-6 treatment upregulated levels of phosphorylated JAK1 (herein pJAK1 refers to pY1034/1035-JAK1) and pY705-STAT3, but not the phosphorylation of JAK2 or Tyk2 (Figure 2A). In contrast, bolus IL-6 treatment failed to stimulate pJAK1 or pY705-STAT3 in IL-6R^−/−^ cells that lacked the receptor. In addition, Med8A-S and IL-6R^−/−^ cells that had been co-cultured with HMC3 exhibited increased pJAK1 and pY705-STAT3 expression (Figure 2A). Subsequently, we evaluated whether treatment with the JAK1/2 inhibitor, ruxolitinib [23], may blockade STAT3-mediated acquired chemoresistance occurring downstream of the cytokine receptors. As shown in Figure 2B, ruxolitinib treatment significantly diminished the levels of pY705-STAT3 of Med8A-S cells stimulated with bolus IL-6. Importantly, ruxolitinib also significantly diminished the levels of pY705-STAT3 in Med8A-S or IL-6R^−/−^ cells induced by co-culture with HMC3 microglia (Figure 2B). Since ruxolitinib effectively reduced pY705-STAT3 expression, we assessed whether combination treatment with vincristine could overcome the resistance of MB induced by co-culture with microglia. As shown in Figure 2C,D, combined treatment with vincristine and a non-toxic dose of 2 µM ruxolitinib effectively overcame the chemoresistance observed in both Med8A-S and IL-6R^−/−^ cells that had been co-cultured with HMC3. These results indicate that targeting JAKs is sufficient to overcome acquired chemoresistance in Med8A-S and IL-6R^−/−^ cells exposed to soluble factors secreted by HMC3 microglia.

### 3.3. The IL-6 Family Cytokines IL-6, LIF, OSM, and IL-11 Promote Acquired Resistance to Vincristine Treatment

**Figure 3:**
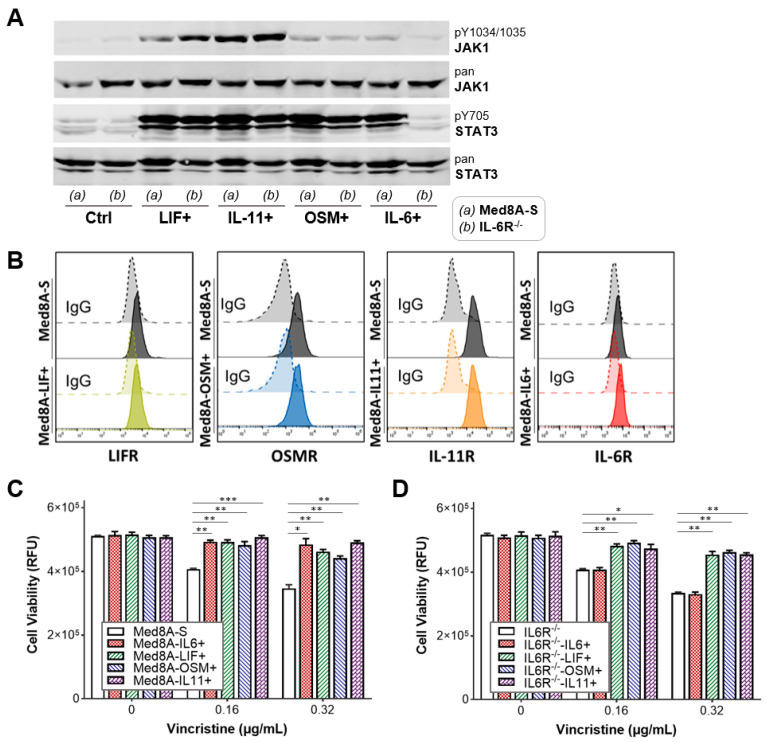
MB cells conditioned with IL-6 family cytokines enhance JAK1 and STAT3 activity and chemoresistance. (**A**) Med8A-S and IL-6R^−/−^ cells untreated (Ctrl) or treated with bolus LIF, OSM, IL-11, or IL-6, and immunoblotted to detect pJAK1, JAK1, pY705-STAT3, and STAT3 expression. (**B**) Flow cytometry assay for cell surface expression of LIFR, OSMR, IL-11R, or IL-6R in Med8A-S and cytokine conditioned cells (LIF, OSM, IL-11, or IL-6), with corresponding IgG controls. Cell viability assay to assess the vincristine sensitivity of (**C**) Med8A-S and (**D**) IL-6R^−/−^ cells not conditioned, or conditioned with IL-6, LIF, OSM or IL-11, as indicated. Plotted is the mean  ±  SD of triplicate assays, representative of 3 independent experiments. *** *p*  <  0.001, ** < 0.01, * < 0.05, two-way ANOVA with Bonferroni’s multiple comparison test.

Stimulatory cytokines that belong to the IL-6 family signal through the common receptor β-subunit, gp130, which results in phosphorylation and activation of downstream JAKs and STAT3 [19]. Hence, we profiled the ability of several members of the IL-6 family cytokines including IL-6, LIF, OSM, and IL-11 to stimulate phosphorylation of JAK1 and STAT3 in Med8A cells. Western blot analysis revealed that Med8A-S cells stimulated with bolus IL-6, LIF, OSM, or IL-11 exhibited enhanced pJAK1 and pY705-STAT3 levels when compared to non-treated cells (Figure 3A). IL-6R^−/−^ cells similarly responded to stimulation with LIF, OSM, or IL-11, but not to IL-6, which was expected (Figure 3A). Flow cytometry analysis revealed that Med8A-S cells expressed detectable cell surface levels of the corresponding receptors LIFR, OSMR, IL-11R, and IL-6R (Figure 3B), a result that supports the ability of the cells to respond to cytokine stimuli.

As we had previously conducted for IL-6 [33], cells were subjected to low dose conditioning with IL-6, LIF, OSM, or IL-11 for four weeks and weaned off this conditioning prior to conducting chemosensitivity assays. We found that both Med8A-S and IL-6R^−/−^ cells conditioned with LIF, OSM, or IL-11 exhibited significant resistance to vincristine treatment in a manner comparable to Med8A-S conditioned with IL-6 (Figure 3C,D). These results further validate the possibility of stimulatory cytokine signaling through their specific α-receptors and common β-subunit to activate STAT3 and induce vincristine resistance in MB cells.

### 3.4. MB Cells Conditioned with One IL-6 Family Cytokine Promote Secretion of Multiple IL-6 Family Cytokines

**Figure 4:**
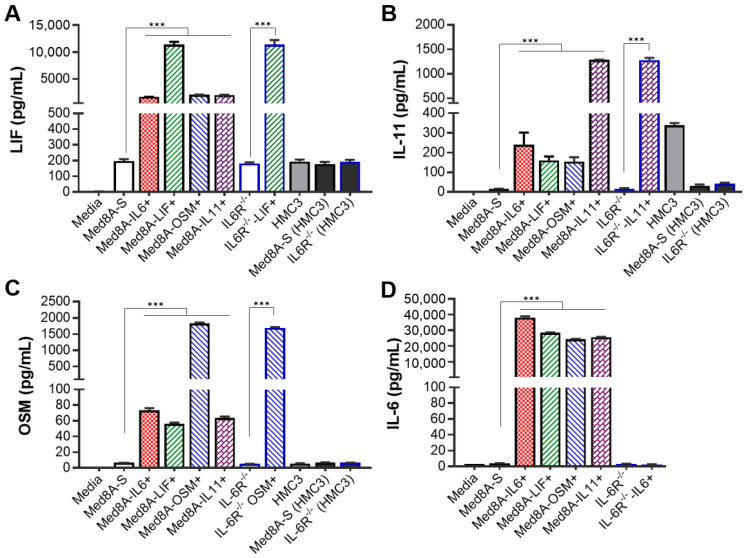
Chemoresistant MB cells secrete elevated levels of LIF, OSM, IL-11 and IL-6. Secreted cytokine analysis for (**A**) LIF, (**B**) IL-11, (**C**) OSM, and (**D**) IL-6 of conditioned media from Med8A-S and IL-6R^−/−^ cells with or without cytokine conditioning (designated as -IL6+, -IL11+, -OSM+ or -LIF+) or exposure to co-culture with microglia (HMC3). The conditioned media of HMC3 cells was also assessed. Plotted is the mean  ±  SD of triplicate assays; *** *p*  <  0.001, ordinary one-way ANOVA with Bonferroni’s multiple comparison test.

We have shown that IL-6-conditioned Group 3 MB cells secreted significant amounts of IL-6 that can act in feedback autocrine signaling [33]. Similarly, Med8A-S and IL-6R^−/−^ cells that had been co-cultured with HMC3 also secreted IL-6 (Figure 1E). To assess whether this phenomenon is also applicable to other cytokines of the IL-6 family, we evaluated the secretion of LIF, OSM, IL-11, or IL-6 in response to exogenous cytokine conditioning of Med8A-S and IL-6R^−/−^ cells as well as upon co-culture exposure to microglia. The results are shown in Figure 4.

Compared to non-conditioned cells, Med8A-S conditioned with a single cytokine secreted significantly higher levels of not only that cytokine, but also of other IL-6 family cytokines. Specifically, we found that Med8A-S conditioned with either LIF, OSM, IL-11 or IL-6, secreted LIF, OSM, IL-11, and IL-6 (Figure 4A–D). This suggests that conditioning with one IL-6 family cytokine is sufficient to promote MB cell secretion of other cytokines belonging to the same family that may act in an additive manner to stimulate downstream JAK/STAT3 signaling.

In addition to IL-6 (Figure 1E), HMC3 microglia also secreted detectable amounts of LIF and IL-11 (Figure 4A,B), suggesting that IL-6, LIF, and IL-11 may contribute to paracrine signaling in the MB tumor microenvironment. However, Med8A-S or IL-6R^−/−^ cells that were co-cultured with HMC3 for three days did not secrete LIF, IL-11, or OSM in excess of the non-co-cultured cells. Collectively, these results suggest that both paracrine and autocrine mechanisms involving IL-6 family cytokines are prevalent in the MB TME that could initiate transformation of chemosensitive tumor cells to chemoresistant variants.

### 3.5. Ruxolitinib Overcomes Chemoresistance of IL-6 Family Cytokine Conditioned MB Cells

**Figure 5:**
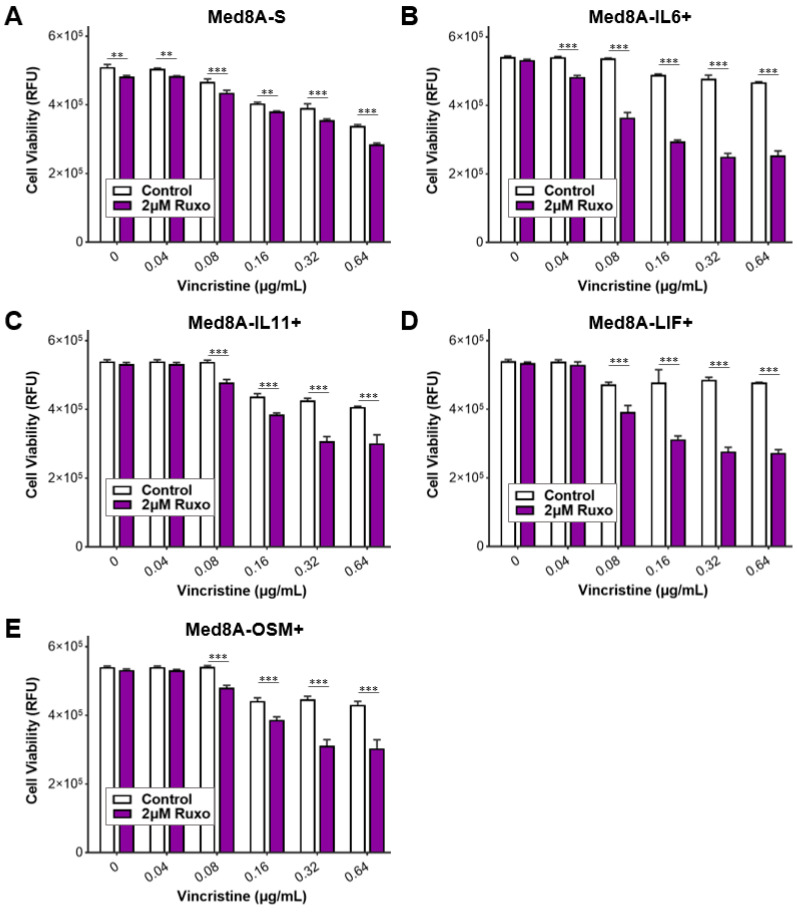
Response of IL-6 family cytokine-conditioned Med8A-S cells to combined treatment with vincristine and ruxolitinib. Cell viability assay to assess the sensitivity of (**A**) Med8A-S, (**B**) Med8A-IL6+, (**C**) Med8A-IL11+, (**D**) Med8A-LIF+, and (**E**) Med8A-OSM+ cells to the indicated concentrations of vincristine with or without 2 µM ruxolitinib co-treatment. Plotted is the mean  ±  SD of triplicate assays, representative of three independent experiments. *** *p*  <  0.001, ** < 0.01, two-way ANOVA with Bonferroni’s multiple comparison test.

**Figure 6:**
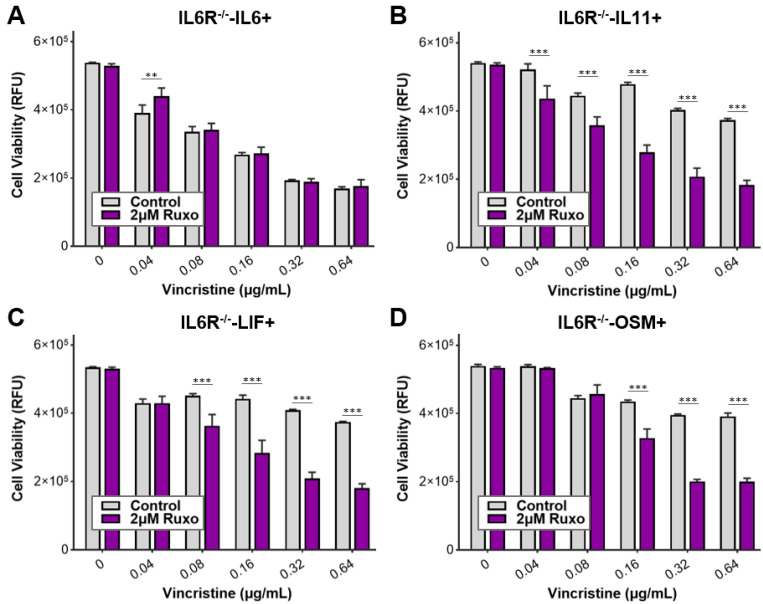
Response of IL-6 family cytokine-conditioned IL6R^−/−^ cells to combined treatment with vincristine and ruxolitinib. Cell viability assay to assess the sensitivity of (**A**) IL-6R^−/−^-IL6+, (**B**) IL-6R^−/−^-IL11+, (**C**) IL-6R^−/−^-LIF+, and (**D**) IL-6R^−/−^-OSM+ cells to the indicated concentrations of vincristine with and without 2 µM ruxolitinib co-treatment. Plotted is the mean  ±  SD of triplicate assays, representative of three independent experiments. *** *p*  <  0.001, ** < 0.01, two-way ANOVA with Bonferroni’s multiple comparison test.

When exposed to soluble factors secreted by HMC3 microglia, Med8A-S and IL-6R^−/−^ cells developed resistance to vincristine treatment that could be overcome with the JAK inhibitor ruxolitinib (Figure 2C,D). In addition, Med8A cells conditioned with IL-6 family cytokines were highly resistant to vincristine treatment (Figure 3C,D). This raises the possibility that JAK inhibition can similarly overcome chemoresistance resulting from IL-6 family cytokine-conditioned MB cells. As shown in Figure 5A–E, combination ruxolitinib and vincristine treatment of Med8A-IL6+, Med8A-LIF+, Med8A-OSM+, or Med8A-IL11+ cells effectively overcame the resistance observed with vincristine alone. In a similar fashion, IL-6R^−/−^ cells conditioned with LIF, OSM, or IL-11 also displayed enhanced sensitivity to combination vincristine and ruxolitinib treatment (Figure 6A–D). These results further confirm that combination treatment with ruxolitinib and vincristine is more effective compared to a single agent treatment in overcoming acquired resistance in MB cells that had been exposed to cytokines for a prolonged period.

### 3.6. Targeting gp130 Mitigates MB Chemoresistance Resulting from Exposure to Microglia

**Figure 7:**
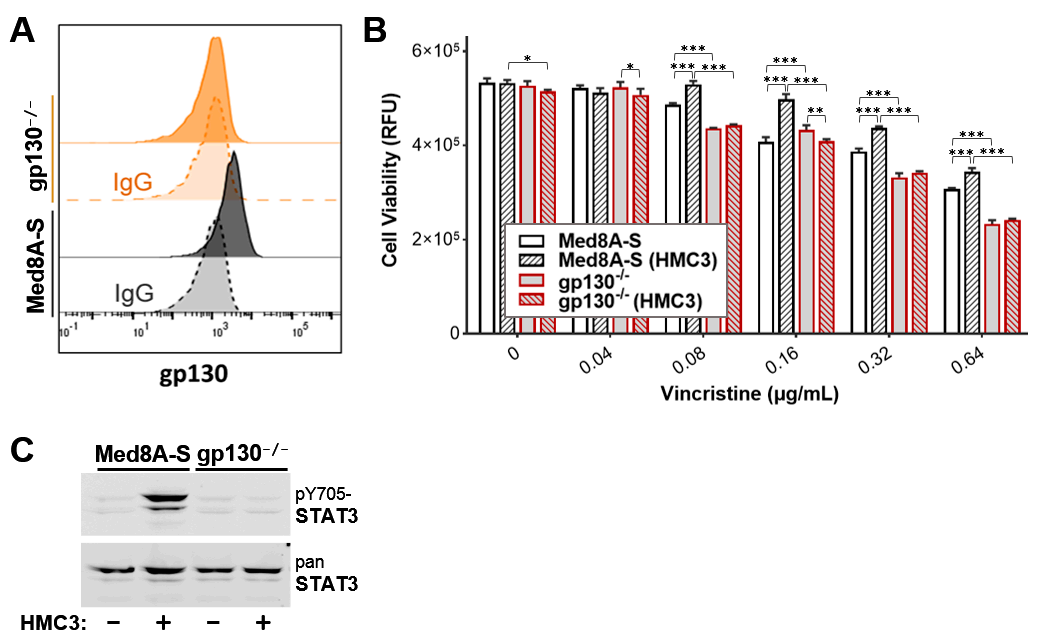
Loss of gp130 prevents microglia co-culture induced STAT3 signaling and chemoresistance of Med8A cells. (**A**) Flow cytometry assay for cell surface expression of gp130 in Med8A-S and gp130^−/−^ cells, with corresponding IgG controls. (**B**) Cell viability assay to assess vincristine sensitivity of gp130^−/−^ and Med8A-S cells with or without HMC3 co-culture. Plotted is the mean  ±  SD of triplicate assays, representative of three independent experiments. *** *p*  <  0.001, ** < 0.01, * < 0.05, two-way ANOVA with Tukey’s multiple comparison test. (**C**) Lysates of Med8A-S and gp130^−/−^ cells untreated or co-cultured with HMC3 were assessed for pY705-STAT3 and total STAT3 by western blot analysis.

The finding that more than one IL-6 family cytokine can promote MB drug resistance suggests that therapeutic targeting of any one cytokine, or its receptor, may not suffice as a strategy to overcome acquired drug resistance. Given that all receptors for the IL-6 family cytokines contain gp130 as the common β-subunit, we evaluated whether targeting gp130 is a viable strategy to overcome chemoresistance. We used CRISPR-Cas9 gene editing to generate Med8A-S cells lacking gp130 expression (gp130^−/−^) (Figure 7A). Unlike the parental Med8A-S cells, gp130^−/−^ cells co-cultured with HMC3 did not develop resistance to vincristine, suggesting that paracrine signaling was attenuated in cells lacking gp130 (Figure 7B). Western blot analysis also revealed that pY705-STAT3 expression was not induced in gp130^−/−^ cells co-cultured with HMC3 (Figure 7C).

**Figure 8:**
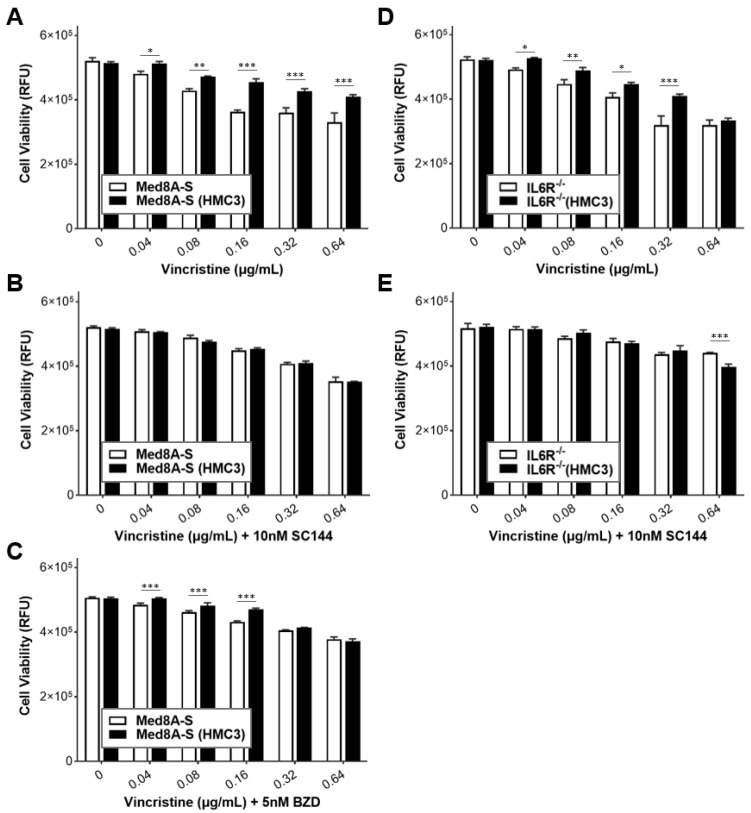
Gp130 inhibitors mitigates microglia co-culture induced chemoresistance in Med8A cells. (**A**) Cell viability assay to assess the sensitivity of Med8A-S cells with or without HMC3 co-culture to vincristine alone, or, (**B**,**C**) in combination with gp130 inhibitors, SC144, or bazedoxifene (BZD), as indicated. (**D**) Cell viability assay to assess the sensitivity of IL-6R^−/−^ cells with or without HMC3 co-culture to vincristine alone, or, (**E**) in combination with SC144. Plotted is the mean  ±  SD of triplicate assays, representative of three independent experiments. *** *p*  <  0.001, ** < 0.01, * < 0.05, two-way ANOVA with Bonferroni’s multiple comparison test.

We evaluated the potential for targeting gp130 using commercially available small molecule inhibitors as a strategy to circumvent drug resistance exhibited by Med8A-S cells when exposed to HMC3 cells. Bazedoxifene is a selective estrogen receptor modulator (SERM) that has been repurposed as a gp130 inhibitor [26], while SC144 is a newer class and potent gp130 inhibitor that effectively blocks activation of downstream signaling including STAT3 [28]. As shown in Figure 8A–C, both gp130 inhibitors enhanced sensitivity to vincristine treatment in Med8A-S cells that had been co-cultured with HMC3. Compared to SC144, bazedoxifene was less effective in overcoming resistance at lower concentrations of vincristine. Combined treatment of SC144 with vincristine was also effective in circumventing resistance of IL-6R^−/−^ cells that had been co-cultured with HMC3 cells (Figure 8D,E). Together, these results suggest that gp130 is a promising target to overcome resistance exhibited by chemoresistant MB cells.

### 3.7. Gp130 Is Essential for Driving Chemoresistance in Other Group 3 MB Cell Lines

**Figure 9:**
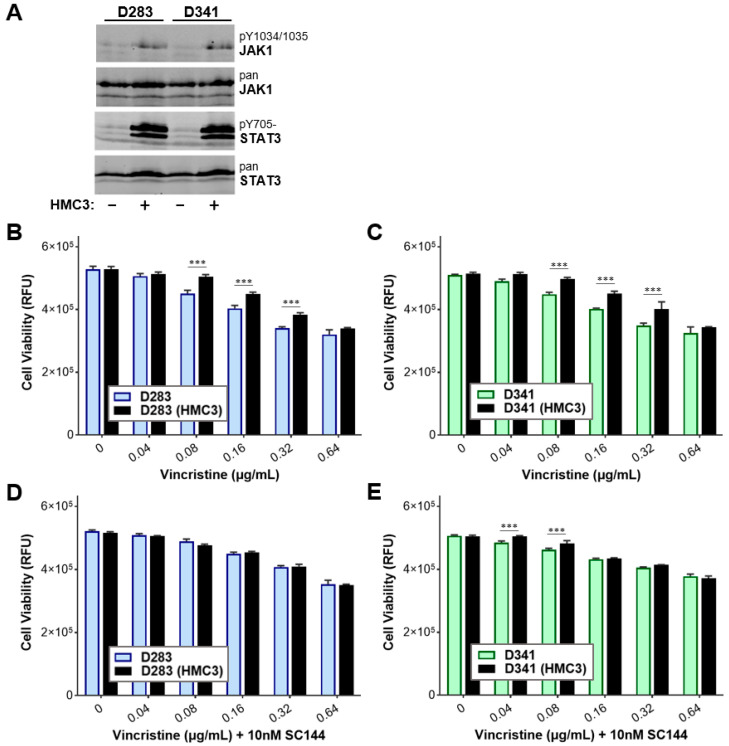
Combination SC144 and vincristine treatment mitigates microglia co-culture induced chemoresistance in D283 and D341 MB cells. (**A**) Lysates of D283 and D341 untreated or co-cultured with HMC3 were assessed for pY705-STAT3 and total STAT3 by western blot analysis. Cell viability assay was conducted to assess the sensitivity of (**B**) D283 and (**C**) D341 cells with or without HMC3 co-culture to vincristine alone, or, (**D**,**E**) in combination with SC144. Plotted is the mean  ±  SD of triplicate assays, representative of three independent experiments. *** *p*  <  0.001, two-way ANOVA with Bonferroni’s multiple comparison test.

Our study demonstrated that gp130 plays an essential role in promoting chemotherapeutic resistance in Med8A cells in response to external stimuli. To evaluate whether this may be a robust phenomenon demonstrated by other Group 3 MB cell lines, we assessed the requirement of gp130 in driving chemoresistance in D283 and D341 cells. STAT3 expression profiling revealed that D283 and D341 cells exhibited increased pY705-STAT3 expression when co-cultured with HMC3 cells (Figure 9A). D283 and D341 cells when co-cultured with HMC3 also demonstrated significant resistance to vincristine treatment (Figure 9B,C). Finally, combination treatment using SC144 to target gp130, in conjunction with vincristine, was sufficient to overcome resistance of D283 and D341 cells that had been co-cultured with HMC3 microglia (Figure 9D,E). Taken together, our data demonstrate that microglia secrete soluble factors that promote chemotherapeutic resistance in Group 3 MB cell lines via STAT3 signaling. Targeting gp130 in Group 3 MB cells holds potential to prevent TME-mediated development of chemoresistant variants of MB and improve the overall therapeutic efficacy when used in combination with vincristine.

## 4. Discussion

The brain TME consists of several cellular components that are thought to play crucial roles in facilitating cellular transformation and tumor progression. Extensive research over the years has highlighted the prognostic importance of immune cells and immunotherapy in cancer biology. Despite the characterization of MB into molecular subgroups, there is limited knowledge about the cellular players present in the MB TME and their contribution to tumor progression.

Our study employed in vitro models to highlight the contribution of microglia in the development of chemoresistance to vincristine in Group 3 MB cells. The chemosensitive Group 3 MB cell line, Med8A-S, when co-cultured with microglia was found to be substantially resistant to vincristine treatment. Additionally, pY705-STAT3 activity was substantially elevated in Med8A-S cells exposed to microglia. This phenomenon is consistent with previous studies demonstrating the association of increased resistance to treatment with increased pY705-STAT3 activity [33,36]. We also demonstrated that while IL-6 conditioning of IL-6R^−/−^ cells failed to promote chemoresistance, microglia co-cultured IL-6R^−/−^ cells exhibited increased resistance to vincristine treatment and elevated pY705-STAT3 activity. This suggests that cells in the TME such as microglia likely secrete a wide range of stimulatory cytokines, in addition to IL-6, which can activate STAT3 and promote the development of chemoresistance. An important implication is that therapeutic blockade of a single cytokine or its receptor may not be sufficient to abrogate STAT3 signaling and acquired chemoresistance in tumor cells that are subjected to multiple cytokine stimuli that exist in the TME. Collectively, these results have paved the way into the exploration of other upstream activators of STAT3 and its contribution to drug resistance in Group 3 MB cells.

The IL-6 family cytokines consist of several cytokines that activate STAT3 and play roles in a multitude of physiological functions [19]. Notably, IL-6 family cytokines in the brain TME contributes to CNS homeostasis and pathogenesis of diseases [30,31,32]. As upstream regulators of STAT3 signaling, we assessed the contribution of some members of this cytokine family, namely IL-6, LIF, OSM, and IL-11, in their ability to promote chemoresistance in the Group 3 MB cells. Our findings showed that both chemosensitive Med8A cells (Med8A-S or IL-6R^−/−^) acquired resistance to vincristine when subjected to exogenous cytokine conditioning for which the corresponding cytokine receptors are expressed. The chemoresistance observed in cytokine-conditioned cells was also associated with increased pJAK1 and pY705-STAT3 levels. These data suggest that IL-6 family cytokines can activate STAT3 in chemosensitive Group 3 MB cells and contribute to acquired drug resistance. More importantly, cells that lack one functional receptor can also develop drug resistance through STAT3 activation in response to other cytokines.

To assess paracrine signaling, we evaluated the secretion of IL-6, LIF, OSM, and IL-11 from microglia as well in chemosensitive MB cells co-cultured with microglia. HMC3 microglia secreted significantly more IL-6 and IL-11 when compared to non-co-cultured Med8A cells, indicating the potential for these cytokines to act as initiating paracrine stimuli to activate STAT3 signaling of MB cells in proximity. In addition, MB cells that had been co-cultured with microglia for three days was found to secrete significant quantities of IL-6, which now constitute a cytokine of tumor cell origin that can signal in autocrine fashion. It is intriguing to consider whether this brief exposure to microglia may be sufficient to kick-start an autocrine signaling loop involving IL-6 family cytokines and further drive the chemoresistance of MB. In particular, we found that Med8A cells that had been conditioned for four weeks with any one cytokine then secreted significant quantities of that same cytokine as well as other cytokines in the IL-6 family. Thus, MB cell exposure to TME cytokines in paracrine signaling may over time result in sustained autocrine signaling and amplification through the secretion of other cytokines, all of which have the potential to sustain and amplify STAT3 activity in a constitutive manner.

Gp130 is a ubiquitous protein that is part of a receptor complex involved in transducing JAK/STAT3 signaling initiated by the IL-6 family cytokines [18,19]. Several studies have demonstrated that blocking gp130 signaling inhibits tumor growth and induces apoptosis in vitro and in vivo in multiple malignancies. Given that gp130 is a common signal transducer for the IL-6 family cytokines, we evaluated the effect of targeting gp130 in Group 3 MB cells to circumvent the acquired chemoresistance. Our findings demonstrate that gp130 expression is required for microglia co-culture induced chemoresistance and promotion of STAT3 activity. In addition, we found that gp130 inhibitors, SC144 and bazedoxifene [26,27,28,37], can be used in combination with vincristine to circumvent the observed chemoresistance induced by co-culture with microglia. Importantly, we used a titrated dose of SC144 or bazedoxifene that was not cytotoxic to Med8A cells when used as a mono-agent, thus potentially minimizing the deleterious side effects of an additional chemotherapeutic on non-tumor cells when used in combination with conventional agents such as vincristine. These striking results further validate the vital role of gp130 in IL-6 family cytokine mediated drug resistance in Group 3 MB cells, which requires STAT3 activation.

In addition to gp130, we also found that targeting IL-6 family receptor downstream signaling using the JAK inhibitor, ruxolitinib [23,38], was similarly effective to circumvent vincristine resistance in MB cells. Of the JAKs that were evaluated, we found that only JAK1 was inducibly phosphorylated in Med8A cells in response to IL-6 stimulation. A non-toxic dose of ruxolitinib effectively blocked IL-6 induced phosphorylation of STAT3, and when used in combination with vincristine, was able to overcome MB chemoresistance, resulting from co-culture exposure to microglia. Notably, a phase 1 trial demonstrated that ruxolitinib is safe to use in combination with multi-agent chemotherapy for pediatric acute lymphoblastic leukemia [24]. Given its safety approved status, and that ruxolitinib is capable of crossing the blood–brain barrier [39], it would be of clinical interest to evaluate ruxolitinib in combination chemotherapy of high risk medulloblastoma such as Group 3.

In conclusion, our in vitro study demonstrated that targeting gp130/JAK/STAT3 signaling attenuates tumor microenvironment mediated chemoresistance in Group 3 MB. Blocking JAK1 activation with ruxolitinib led to diminished pY705-STAT3 activity and rendered chemoresistant Group 3 MB cells more susceptible to vincristine treatment. The evidence suggests that JAK1 might be a potential target to combat drug resistance in Group 3 MB. Additionally, prolonged exposure to stimulatory cytokines contributed by cells such as microglia present in the brain TME can promote chemoresistance via a paracrine signaling mechanism. Future studies could investigate the role of other cells in the brain TME that contribute to the development of drug resistance in Group 3 MB cells. Understanding the constituents of the subgroup specific MB TME can also yield benefits in understanding the pro- or anti-tumor effects that affect disease progression as well as identify novel therapeutic strategies to counter MB that are refractory to conventional therapies [40]. Loss of gp130 resulted in diminished pY705-STAT3 activity and prevented acquired drug resistance in chemosensitive MB cells exposed to microglia. Notably, inhibiting gp130 signaling with targeted inhibitors such as bazedoxifene or SC144 in combination with vincristine can effectively overcome chemotherapeutic resistance. The multi-faceted nature of gp130 makes it a promising therapeutic target for the treatment of Group 3 MB tumors. Future studies are required to determine the efficacy of these inhibitors as a single agent and in combination with cytotoxic drugs in an in vivo setting.

## Data Availability

Data is contained within the article.
